# Referrals to Peer Support for Families in Pediatric Subspecialty Practices: A Qualitative Study

**DOI:** 10.1007/s10995-025-04062-1

**Published:** 2025-01-29

**Authors:** Alex Kobrin, Olivia Chan, Emily Crabtree, Joe Zickafoose, Amy Wodarek O’Reilly, Edward Schor, Holly Henry, Allison Gray

**Affiliations:** 1 Mathematica, P.O. Box 2393, Princeton, NJ 08543–2393 USA; 2 Stanford University, 1835 Bay Laurel Drive, Menlo Park, CA 94025 USA; 3 Lucile Packard Foundation for Children’s Health, 400 Hamilton Avenue, Suite 340, Palo Alto, CA 94301 USA

**Keywords:** Parent peer support, Children with special health care needs, Pediatric subspecialty care, Care coordination, Caregiver

## Abstract

**Introduction:**

Referrals to peer support (PS) can help families of children with special health care needs in providing emotional support, reducing feelings of stress and anxiety, and improving the care experience. This study aimed to gain providers’ perspectives about PS referrals for families of children with special health care needs, including their perspectives on logistics of, barriers to, and facilitators of making referrals as well as the perceived impacts of PS referrals.

**Methods:**

This study builds on a 2022 survey of California pediatric subspecialists about the value and challenges of PS. The study team conducted 20 semistructured interviews with people from pediatric subspecialty practices in California and used a priori themes derived from the interview protocol to develop a codebook, code interview transcripts, conduct a thematic analysis, and summarize findings.

**Results:**

Respondents offered a variety of PS referrals inside and outside their institutions, tailoring referrals to each family’s needs and preferences. Social workers and family liaisons were most commonly responsible for making PS referrals. Respondents found that care team collaboration and ease of sharing information about PS resources among colleagues facilitated the referral process. Respondents noted a need for more PS resources, including funding, education, and the need for a network where providers can identify PS resources.

**Discussion:**

Encouraging PS program information-sharing within and across organizations could help connect more families to PS services. Future research should assess families’ experiences with PS referrals and services to understand approaches that can best meet their needs for information, instrumental, and emotional supports.

**Supplementary Information:**

The online version contains supplementary material available at 10.1007/s10995-025-04062-1.

## Significance

What is already known on this subject?

What this study adds?

Referrals to peer support can help ease feelings of stress and anxiety for families of children with special health care needs and improve their understanding of diagnosis and treatment. Most California pediatric subspecialists support the idea of making such referrals.

This study identifies factors that providers consider when referring families to peer support, including logistical, cultural, and familial circumstances and functional status. There appears to be considerable agreement among subspecialty practice staff about the value of peer supports for families and the need to increase its availability.

**Supplementary Information:**

The online version contains supplementary material available at 10.1007/s10995-025-04062-1.

## Introduction

Nearly one in five children in the United States has a special health care need (Maternal & Child Health Bureau, 2022), with diagnoses encompassing physical, developmental, behavioral, and emotional conditions (Maternal & Child Health Bureau, 2023). Managing special health care needs and navigating the system of care can be incredibly stressful for families and can lead them to feel socially isolated (Baker & Claridge, 2022). Research has found that connecting caregivers of children with special health care needs (CSHCN) with others who have similar experiences helps them ask questions about care and understand what to expect about their child’s condition, develop advocacy skills, reduce their sense of isolation, and give them hope for the future (Chakraborti et al., 2021; Hall et al., 2015; Hughes, 2015). These connections are referred to variously as caregiver peer support, parent-to-parent support, family-to-family support, or peer support (PS).

Pediatric subspecialists and their practice staff often have ongoing relationships with families of CSHCN with some of the most intensive needs. Consequently, they are in a strong position to identify families who could benefit from referrals to PS (Schor & Fine, 2022). Referrals to PS can be formal or informal and include referring caregivers to parent support groups, virtual parent-to-parent resources, or individual peer mentors who have experienced similar situations and, ideally, have received some training for that role (Bray et al., 2017; Tully et al., 2017). These resources can be inside or outside the medical care setting (Chakraborti et al., 2021).

Although families value PS, there is little known about how referrals to these resources are made (Schor & Fine, 2022). This study aims to examine referral processes and how subspecialists help people access PS. It follows up on findings from a 2022 survey of pediatric subspecialists in California, which aimed to understand the extent to which the subspecialists provided PS referrals to caregivers of CSHCN (Schor et al., 2025). Many subspecialists viewed PS referrals favorably, but they were not always familiar with available PS resources. The extent to which they or their practices provided referrals was affected by their knowledge of resources, the time available, staffing, and institutional support. This follow-up study was designed to build on those findings and better understand the subspecialty practices’ processes, barriers, and facilitators regarding PS referrals. This research aims to inform future efforts to improve referrals and access to PS.

## Methods

**Overview.** The researchers conducted semistructured qualitative interviews of staff at pediatric subspecialty practices across the state of California from August to November 2023. The methods used to conduct this research align with the Consolidated Criteria for Reporting Qualitative Research (COREQ) checklist.

**Interview Protocol.** The study team developed an interview protocol (Appendix 1) that contained branching logic based on a respondent’s answer to certain questions, allowing interviewers to tailor conversations depending on respondents’ specialty, role, and involvement with PS referrals.

**Institutional Review Board (IRB) Review.** The team conducted the research in accordance with prevailing ethical principles; the Health Media Labs IRB approved this study before data collection began.

**Respondent Recruitment.** The team primarily recruited from people identified by pediatric subspecialists who responded to the 2022 survey. In that survey, respondents volunteered the name and contact information of someone the researchers could contact in the future for more information about PS referrals in their practices. The aim was to interview 20 people from pediatric subspecialty practices in California based on the numbers needed to reach thematic saturation and to achieve diversity of specialty and practice institution.

The team began with a purposive sampling approach to achieve perspectives from different specialties and institutions and contacted 72 people from the 2022 survey; 15 agreed to be interviewed. Based on contacts suggested by the first 15 respondents, the team used snowball sampling to recruit another 5 respondents to achieve diversity.

**Conducting Interviews.** Interviews occurred from August to November 2023 and lasted about 45 min via video call. As compensation, respondents could receive a $50 Amazon gift card or a $50 donation to the Special Olympics. All respondents received and reviewed consent language before the interview and verbally consented to participate. Each interview was conducted by an experienced qualitative researcher who recorded the interviews when the respondents provided verbal consent to record. A professional transcription service transcribed the recordings for coding.

**Analysis.** The team identified a priori themes^1^ from the interview protocol to develop a codebook and used NVivo,^2^ a qualitative coding software, to code and analyze the data and conduct a thematic analysis by summarizing findings across respondents for each code. The team piloted the codebook with one interview transcript and refined the codebook to ensure interrater reliability. Two team members each coded half of the 20 interview transcripts, and the study lead conducted quality assurance of all coded transcripts to ensure coding consistency. To give a sense of the prevalence of responses among respondents without overemphasizing counts in this qualitative study, many findings are framed using the following terminology: all (100%), many (50–99%), some (1–49%), or none (0%) of the respondents.

## Results

The final study sample included 20 people working in a variety of roles [Table 1], with a majority (80%) being non-physicians; interviewees were primarily social workers because the study team requested that subspecialist participants in the original study identify the person in their practice responsible for PS referrals for a follow-up interview. Both physicians and non-physicians interviewed had experience making PS referrals, although most physicians said they held a more supportive role in the PS referral process. The study also included perspectives from various pediatric subspecialties [Table 2] across nine hospitals in California; the most common specialties were hematology/oncology/neuro-oncology, neonatology, and nephrology/neurology. The team observed thematic consistency across respondents, and key themes from the qualitative interviews are outlined below.

**Table 1:** Count of respondents by role

Role	Count (%)
Physician	4 (20%)
Non-physician	16 (80%)
Social worker	13 (65%)
Parent liaison	2 (10%)
Care coordinator	1 (5%)

**Table 2:** Count of respondents by subspecialty

Subspecialty	Count (%)
Neonatology	3 (15%)
Other subspecialties	17 (85%)
Pediatric hematology/oncology/neuro-oncology	9 (45%)
Pediatric nephrology, neurology	3 (15%)
Pediatric transplant hepatology	1 (5%)
Developmental behavioral pediatrics	1 (5%)
Pediatric pulmonology	1 (5%)
Pediatric endocrinology	1 (5%)
Pediatric medical genetics	1 (5%)

Subspecialties are staffed by both physician and non-physician respondents

### Referral Process

**Evaluation of Need.** Nearly all respondents depended on structured psychosocial assessments and clinical judgement to identify families’ need for PS. The most cited factors used when considering referrals were the following:

- *Psychosocial needs*. Respondents said they referred families that exhibit heightened fear, stress, anxiety, or challenges coping with a diagnosis or treatment plan.
- *Limited resources and social support*. Respondents noted that they were more likely to refer families with limited family or community support, language barriers, less experience navigating the health care system, and lower incomes.
- *Diagnosis and needs*. Some respondents said they discuss PS opportunities with every family they meet with as part of their standard workflow for a first encounter. In other practices, staff identify families facing rare diagnoses, complex or higher acuity medical needs, surgeries and transplants, and challenges understanding a new diagnosis.
- *Timing*. Respondents noted that some medical situations might require immediate decisions by caregivers who then do not have sufficient time to be connected to PS. Some practices do not offer PS initially until they feel the caregivers are ready to connect with others outside their immediate support system. “It’s never something that I say in the first sentence because there’s too much going on,” one social worker remarked. “They have to settle a little from that crisis of hearing their child has the thing that they fear the most in life…”

Many respondents noted that caregivers often expressed interest on their own and asked to be connected to PS. Many asked to be connected to “experienced” families (that is, families that have dealt with similar diagnoses and can offer advice and emotional support).

**Making PS Connections.** Beyond knowing what PS resources exist and evaluating a families’ need for support, respondents considered which resources would be a good fit for each family. Many noted that families preferred diagnosis-specific support groups to connect with others experiencing similar diagnoses and treatments. Some respondents mentioned PS programs with specific groups for each family member (such as parents and siblings). Respondents considered families’ preferences when making referrals. For example, some families preferred to be matched with a one-on-one mentor, and others preferred a group PS setting (both in person and virtual). Several respondents noted that, although they do not refer to resources on social media, some families find PS groups and forums on social media through their own online searches. Some respondents said that external organizations provided online PS, including virtual meetings (often over Zoom); external organizations referenced include the Down Syndrome Association, Pediatric Brain Tumor Foundation, Childhood Cancer Foundation, CureDuchenne, and various local family resource centers. Although some families appreciated the flexibility of virtual options, one respondent shared that one of the families that they worked with reported that this method was not as helpful as in-person PS.

The process of connecting families to PS was described by respondents as “informal,” lacking a documented or standard procedure for all practice staff to follow. To ensure compliance with the Health Insurance Portability and Accountability Act (known as HIPAA), staff seek consent from families before sharing their contact information with a resource. Some practices use signed release forms, but many obtain verbal consent. When connecting families to PS resources, staff sometimes share the family’s contact information directly with the resource but often leave it up to the family to make the initial contact.

**Matching Families to Peer Mentors.** Many respondents said that they introduce families for peer mentorship, a type of PS that offers one-on-one connections between families. Making an appropriate match is particularly important when connecting mentee and mentor caregivers. Respondents noted the following factors they consider when making a match:

- *Diagnosis—timing and condition.* Respondents often aimed to match families who have children with similar diagnoses and courses of treatment. They typically selected caregivers who are further along in their child’s diagnosis and treatment as the mentor, which allows mentee caregivers to receive advice from a family that has more experience with the diagnosis and care plan.
- *Socioeconomic, cultural, and linguistic background.* Some respondents noted that families can feel more comfortable when matched with those of similar backgrounds. Several respondents said that pairing Spanish-speaking families allowed them to receive advice in their primary language and enabled them to better understand the condition and care plan. Respondents also noted that families’ socioeconomic circumstances can affect their access to resources, causing differences in their ability to manage their children’s conditions. Mentor caregivers who have faced such difficulties might be more helpful to families who are newly navigating systemic challenges.
- *Patient age.* Some respondents matched families of similar-age children.
- *Location.* Some respondents matched families that live in the same region so that local mentors and mentees can connect in person and share community-specific resources.
- *Coping and understanding of condition.* Respondents emphasized the importance of selecting mentors who are positive, demonstrate good coping skills, and have an accurate understanding of the diagnosis and care. They cautioned against matching mentee caregivers with caregivers who mistrust the health system or who had a negative care experience because this can oppose the medical guidance mentees receive.
- *Training.* Respondents noted a range of training requirements for peer mentors, ranging from specific PS training at external organizations, general training for volunteer staff at the practices, to no formal training for this role.

**Documentation and Follow-Up.** Nearly all respondents recorded internal and external referrals in the patient’s electronic health record. Many providers did not bill for the time spent providing referrals; several social workers noted that providing referrals is a component of their salaried role.

After providing a PS referral, many respondents followed up with caregivers informally, often by checking in during families’ subsequent visits to see whether they connected with the resource or mentor and how the interaction went. Respondents emphasized the importance of encouraging families’ agency, noting that it should be a family’s decision to pursue PS. As one social worker said, “We can’t make anyone do anything, that’s just not our role. But we empower people to try to get support and we encourage that, and then they take the ball, or they don’t.”

### Referral Services

**External Services.** Nearly all respondents referenced external PS resources to which they regularly referred families. Common external resources for PS included national organizations that provide educational and PS connections such as the Pediatric Brain Tumor Foundation, Autism Speaks, American Diabetes Foundation, Epilepsy Foundation, and the Center for Rare Diseases. Some respondents preferred referring families to local community-based organizations. Many of the external resources referenced were specific to a diagnosis. Some respondents referred families to family resource centers or networks that had their own processes for assessing families and connecting them with educational, financial, and emotional, or peer supports. Several respondents referred families to family camps or weekend retreats held by foundations that also offered social events and PS groups tailored to parents and caregivers, siblings, and patients.

Some respondents said that their practice relies on PS resources that have been long established at their institution and in their respective pediatric subspecialty field. Social workers noted that their knowledge of community resources is a critical aspect of their role; they learn about some resources when they are onboarded to their role and add to that knowledge through internet searches, networking, and experience working with families.

Some respondents mentioned that organizations have reached out to their practice to market their programs and services. Respondents expressed wariness about partnering with unfamiliar organizations. They emphasized the importance of vetting organizations to ensure they did not provide inappropriate medical advice or were not solely selling a service.

**Internal Services.** Nearly half the respondents noted that their practices offer some form of internal PS services in group settings or one-on-one sessions. Some practices offer formal PS groups facilitated by social workers or psychologists that meet regularly. One respondent said that their PS groups have a topic for each meeting, such as “coping, adjusting to new diagnoses…or self-care for caregivers.” Other practices offered drop-in group sessions in which caregivers can meet other families and discuss their experiences. Some practices offer one-on-one PS services for families. For example, one practice assigned “parent liaisons” to each family with a child in the neonatal intensive care unit to help families navigate their child’s care, connect them with resources, and provide emotional support. Several practices connected families with peer mentors, as discussed above.

Respondents also spoke about social events, such as family days, designed as an opportunity to meet other families. Several respondents said they halted PS groups during the COVID-19 pandemic, and not all have been restarted. Many PS groups were previously held in person and have transitioned to virtual meetings.

### Barriers and Facilitators

Respondents noted the following barriers and challenges to making PS referrals:

- *Cultural and language barriers.* Respondents explained that some families are not comfortable discussing their experience with strangers, especially when services are not offered in their preferred language.
- *Lack of established, reliable PS services.* Several respondents noted a lack of available or reliable PS services in their communities. Although some respondents mentioned local family resource centers, many expressed a desire for a system to keep track of available PS resources. One respondent suggested that organizations could hold educational meetings to make providers aware of available PS resources.
- *Limited funding for PS programs.* Some respondents pointed to limited funding for organizations that offer PS, especially in the wake of the COVID-19 pandemic.
- *Limited time to make referrals.* Several respondents noted that they do not have adequate time in their day to make referrals or to follow up with families.
- *Need for more peer mentors.* Many respondents wanted more peer mentors in their institutions but recognized that there are not enough mentors to connect with all the families who could benefit from mentorship, particularly as the peer mentor position is often unpaid.
- *Logistical challenges.* Respondents saw that it can be difficult for families to attend group PS programs because of scheduling barriers such as finding childcare, travel time, competing daytime responsibilities, and taking time off from work.

Respondents noted multiple characteristics that improved their ability to provide PS referrals:

- *Care team collaboration.* Some respondents said that strong collaboration between various members of the care team—such as holding regular morning huddles or divvying responsibilities across team members—can facilitate successful referrals.
- *Dissemination of information.* Staff provided examples of strategies they use to make families aware of PS resources, including flyers, handouts, emails, and, in one case, a QR code on the back of their badge.
- *Making the introduction.* Respondents expressed that sufficient time to introduce families to PS services and to facilitate follow-up can help lead to successful connections between families and PS resources.
- *Relationship with external PS staff.* Some respondents described how relationships with staff at external PS programs can facilitate the referral process because it gives them a key person at the organization that they can communicate with. One respondent described how a staff member from an external resource regularly comes to their hospital to share resources and introduce themselves to families who are considering PS.
- *PS structure.* Some respondents noted that PS groups with a social and therapeutic element encouraged participation. One respondent said that having a PS group run by multidisciplinary teams (including a psychologist and social worker) ensured that all the needs of families participating could be met.

### Referral Outcomes

Respondents said that referrals to PS can be a valuable resource to families and to providers.

**Impact on Families.** Several respondents mentioned that being connected to another family who has gone through a similar experience helps families. One respondent said, “to have…a parent that is dealing with a diagnosis-specific issue. [Support from another family] is irreplaceable.” Respondents have seen these connections reduce feelings of isolation that families experience when they have a child with special health care needs and noted that PS can validate families’ experience and concerns, which can lead to better stress management and decreased anxiety. Respondents said they have seen the connections transform people’s fears into hope. One respondent noted that these connections are “a very, very important part of helping people stay as healthy as possible.” Many respondents indicated that PS improves caregivers’ skills and confidence as well as families’ ability to process medical information, particularly for non-English speakers.

**Impact on Providers.** According to one respondent, PS often complements the medical advice that providers offer. A social worker explained that referring to PS is helpful because her caseload is large; although she is not always able to provide the level of emotional support that families need, peer mentors take on this emotional support role.

Nearly all respondents noted that, in the future, they would like to see their peer referral networks expanded. Nearly half of respondents said that an online resource hub or established referral network should be created to facilitate the referral process. According to one respondent, providers are often unaware of the extent of PS resources available, and having an accessible hub could help improve awareness.

Respondents mentioned several PS services that they would like to see offered or expanded in their practice or community, including the following:

- Multilingual PS services
- Adolescent and young adult-specific support groups
- Virtual support groups (for example, via Zoom)
- Diagnosis-specific support groups

Finally, one respondent said that they want to hear from families who have received PS services to learn about their experience with and perspectives about PS. Providers would find this information useful to help make future referrals.

## Discussion

Respondents said that PS referrals for families of CSHCN are valuable, offering families similar benefits as those noted in the literature, such as improved psychosocial outcomes and efficacy in caring for the CSHCN (Chakraborti et al., 2021; Hall et al., 2015; Hughes, 2015). As study participants provided perspectives of pediatric care institutions, they also emphasized the value of these referrals for providers. Similar to findings in Schor & Fine, 2022, subspecialists and practice staff in this study are frequently connected with families of CSHCN, understand their medical and social needs, and understand the availability of peer mentors, making them well positioned to provide PS referrals. Routinely integrating offers of PS referrals into regular care might be helpful for simplifying workflows and for meeting families’ needs for information and support. There are also opportunities to standardize the PS matching process, as identifying and forming matches between mentees and mentors for families is currently largely informal and based on clinical judgement.

Regardless of their role or subspecialty, respondents identified similar referral process characteristics, concerns, and successes. Social workers, who were the largest subgroup of respondents, were able to provide more exhaustive lists of the external and internal programs to which they frequently refer families compared with respondents in other roles, likely because knowledge about referrals falls directly within their care responsibilities.

Although providers generally agreed on the importance of PS referrals and expressed positive views of and experiences with PS, respondents expressed that referrals should not have a one-size-fits-all approach. Providers should consider the following when making referrals:

**Matching Families to PS Services.** Evaluating and making the *right* connections for each family is critical to a successful outcome. Beyond this, several respondents said that PS services should be properly vetted to ensure they will be a reliable and supportive resource to families.

**Timing.** Often, families are initially overwhelmed by a diagnosis, and care should be taken to ensure that the referral is introduced to them at a time that will help the family and not add additional burden.

**Desire for Referral.** Not all families want a referral to PS. Some families prefer to keep their journey private.

Strong care team coordination and adequate resources such as staff time, education, and funding for PS programs could help facilitate successful PS referrals.

**Limitations.** Although this study provides useful insights, several limitations could affect the representativeness of our findings. We observed thematic consistency, but the sample of respondents was limited and the opinions and experiences of pediatric subspecialty hospital staff that volunteered to participate might differ from those who were unable or unwilling to contribute their perspectives. Our sample overrepresents certain institutions in which more people were interested in participating. In addition, we were not able to interview respondents from every pediatric subspecialty, and there may be specialty-specific considerations for PS referrals.

**Future Directions.** Many respondents expressed a desire for the expansion of PS services in the future. Efforts to create a network of PS providers across practices in California could be beneficial to the future of PS referrals, as lack of awareness of reputable PS resources is a current barrier to providers who are offering these referrals.

Although this qualitative research provides an understanding of the landscape of PS referrals for providers, the community may benefit from further research on the topic to understand how PS referrals affect families directly and whether families are able to access the PS they need. Surveying or interviewing families who have received such services could provide valuable insight to providers for ways to improve PS referrals and availability and types of PS services to meet families’ needs.

## Supplementary Information

Below is the link to the electronic supplementary material.

Supplementary file1 (DOCX 26 KB)

## Data Availability

Not applicable.

## Footnotes

1 A priori themes used for analysis included background and role, key staff, external peer support services, internal peer support services, start of referrals to peer support services, resource introduction, example case, referral process, impact to families, barriers/challenges, successes/facilitators, champions, and perceived value and future directions.

2 https://help-nv.qsrinternational.com/20/win/Content/about-nvivo/about-nvivo.htm
