# Supplementary material for: Referrals to Peer Support for Families in Pediatric Subspecialty Practices: A Qualitative Study
Source: Matern Child Health J. 2025 Jan 29;29(2):280–6. doi: 10.1007/s10995-025-04062-1 (PMC11821679; doi:10.1007/s10995-025-04062-1)
Supplement: Supplementary file 1 — Supplementary file1 (DOCX 26 KB) [file 10995_2025_4062_MOESM1_ESM.docx]

**Appendix 1.**

**Caregiver Peer Support Interview Protocol**

*Introduction and informed consent (all respondents)*

Thank you for taking time to speak with us today. We invited you to participate in this interview because [either you or a colleague provided your contact information during the 2022 California Survey of Parent Support/your colleague provided your contact information as someone who may be knowledgeable about caregiver peer referrals]. My name is … [name/role]. I’ll start by sharing a bit of information about our project, and then I can answer any questions you might have before we begin.

[Author] supports activities aimed at improving the system of care for children with special health care needs, including those with complex medical needs. [Author] is working with [Author] to explore the landscape of caregiver peer support for families of children with special health care needs.

Caregiver peer support, often referred to as family-to-family or parent-to-parent support, offers connections to other families with similar experiences to provide personal, ongoing support.

We want to learn about how referrals for caregiver peer support for families of children with special health care needs occur in pediatric subspecialty practices across California and identify any factors that might facilitate or impede the referral process. Referrals can range from informal connections to individual patient caregivers, to more formal referrals to established peer support programs (individual and group) within a clinic, hospital, external community-based organization, or social media platform.

Participation in this study is voluntary, and you may decline to participate or decline to answer any specific questions throughout the interview. There will be no consequences if you decide not to participate. There are no known risks to participating in this study. Your participation will help inform the Foundation’s work related to caregiver peer support.

We will not use your name or the name of your organization in any report or publication from this study. Your identity will be strictly confidential, and your comments will never be connected to you. We will combine the information we learn from you and other respondents, to be shared in a written report. Interview recordings, notes, and transcripts will be stored in a project folder that is password protected and can be accessed only by our research team. Deidentified data for this study may be archived for future use by other researchers. If you have any questions or concerns, you can ask me now or contact our project director, [Name] at [Phone] or [Email].

Today’s interview will last about 45 minutes. To better focus on our conversation, I would like to record the interview, so it can be transcribed for our notes. We will share the transcripts with the Foundation and to a transcription vendor, but we will remove your name and title from the transcripts before sharing them.

Is it OK with you if we record the call?

*[Interviewer: If NO, inform the participant that we can conduct the interview but will not record.]*

*[If YES, begin recording.]*

Do you agree to participate in the interview?

*[Interviewer: If NO, thank them for their time and end the call.]*

We may have follow-up questions as we analyze the results of the interviews. Would you be ok with being contacted by email if we have any follow-up questions for you?

*[If NO, make a note that we may not follow up with the respondent in the future.]*

Do you have any questions before we start?

| **Question** | **Probe** | **Respondent** | |
| --- | --- | --- | --- |
| **Background**   1. **Respondent’s background** | | | |
| To start, I’d like to learn a bit more about your role at [organization name]. | | | |
| 1. According to our notes, you are [position title]. Would you please tell us a bit about your patient care roles and responsibilities? | a. How long have you been in your role? | **[ALL]** | |
| 2. You mentioned your practice [does/does not] provide caregivers of children with special health care needs with referrals to peer support. Is this accurate? | a. Referrals can be formal or informal and can involve referring caregivers to parent support groups, virtual parent-to-parent resources, or individual caregivers (peer mentors) who have experienced similar situations, to ask questions about care and what to expect.  b. [If respondent says they do not offer referrals:] Has your practice offered any type of referrals or connections to peer support programs or to individual patient caregivers? Referrals can be formal or informal and can occur within the practice, a hospital, a community-based organization, or a social media platform.  [If they offer referrals, go to B1. If they do not, skip to DO NOT OFFER section.] | **[ALL]** | |
| **Background**   1. **Organization’s or institution’s background** | | | |
| Now, I would like to ask you about referrals to caregiver peer support at your practice. | | | |
| 1. Does your practice provide referrals to peer support within your clinic or institution, or do you refer to an outside organization? | a. When did your practice start offering referrals?  b. [If they refer to an outside organization:] What organization do you refer to? Do you know how you or your practice learned about this organization?  c. [If they offer referrals internally:] Is the peer support service or program offered institute-wide or within particular clinic settings?  *i. [If institute-wide:] Which department houses the service or program?* | **[OFFER]** | |
| 2. Are you personally involved in the peer support referral process? |  | **[OFFER]** | |
| **Open-ended case**  Can you recall a specific situation when you made a referral to caregiver peer support? Would you tell me about it briefly? What was the condition of the child that prompted consideration of a referral to caregiver peer support? What nonmedical (social, emotional, or family dynamic) factors were at play? In what setting did you make the referral? Did the referral occur before or after a diagnosis was made? | | | |
| **Referral process**   1. **Evaluation of need** | | | |
| **[If example case is provided:]**  In the context of the example you provided, I would like to ask you about the processes in place that helped you provide caregiver peer support referrals at your practice.  **[If no example could be provided:]**  Now, I would like to ask you about the processes in place to provide referrals to caregiver peer support at your practice. | | | |
| 1. When does your practice consider referring a caregiver to peer support? | a. What factors would lead you to consider a referral for peer support (for example, certain conditions or prognoses, family stress level, or other social or emotional needs)?  b. Is there a formal or informal process at your practice to evaluate whether a family should be referred or connected to a peer support?  c. What does that process look like?  d. How consistently are peer support services offered? | **[OFFER]** | |
| 2. Who initiates the referral process? | a. The patient’s family? The provider or a staff member at the practice? Someone else?  b. Who at your practice is involved in *completing* a referral to peer support? This could include family mentors or liaisons, physicians, social workers, or others.  *i. Would the people involved in peer support referrals differ in an inpatient setting as compared with an outpatient setting?*  c. [If the provider or staff initiates the process:] Do families ever ask to be connected to other experienced families? If so, in what circumstances? | **[OFFER]** | |
| **Referral process**   1. **Awareness and choice of peer support opportunities to refer to** | | | |
| 3. How does your team decide where to refer families? | a. Are there certain services or programs that your team prefers to refer families to? Are these within your practice or external (or a mix of both)?   1. *[If internal:] Would you tell me more about where you refer to? For example, to an established parent mentor program or an informal connection to an individual family member?* 2. *[If external:] Would you tell me more about which external resources you refer families to (for example, community-based organizations, condition-specific organizations, or family resource centers)?* 3. *[If both:] Are any policies in place that define whether you should provide an internal or external referral?*   b. Are these services provided by paid staff or volunteers?   1. *Are people who provide peer support services required to complete any training?*   c. Can you describe a typical encounter between a peer mentor and a caregiver? | | **[OFFER]** |
| 4. [If peer mentors are used:] Can you tell us how families are matched to peer mentors? | a. What factors do you consider when matching families with peer support/peer mentors? | |  |
| 5. In what format are peer support services provided (virtual or in person; one-on-one or group support)? | a. Do your decisions on where to refer families differ based on whether the patient is in an inpatient versus an outpatient setting? | |  |
| **Referral process**   1. **Handoff and care coordination** | | | |
| 6. Would you briefly describe how the referral process is completed? | a. What type of handoff does your team provide to internal referrals? To external referrals?  b. How are peer support referrals documented? Are they documented in the electronic health record?   1. *Who documents these referrals?*   c. Do you or your team follow up with families you’ve referred for peer support?  *i. What does this follow-up look like?* | **[OFFER]** | |
| **Assessment of referral process** | | | |
| In this last section, I want to ask about how referrals to caregiver peer support have affected your patients and their families, your colleagues, and your practice. You can keep in mind the example you provided earlier, but we would also like to hear about your broader experience with these referrals. | | | |
| 7. How do you think a referral to peer support can impact families? | 1. Processing medical information (is information provided useful or inaccurate; does it help family understanding of disease or condition; encourage or discourage following provider recommendations?) 2. Emotional support and stress management? 3. Assistance connecting to social services and other community resources 4. Improving caregiving skills and confidence 5. Improving satisfaction with care 6. Improving health outcomes | **[OFFER]** | |
| 8. What feedback have you received from families that have been referred to these services? | a. Were families satisfied or unsatisfied with the services provided?  b. Did they find these services valuable? What did they find valuable? (for example, understanding of disease, emotional support, sense of community)?  c. Do families have opportunities to provide feedback on the peer support services provided? If so, how do they provide the feedback? | **[OFFER]** | |
| 9. What do you consider the major barriers to making referrals for peer support? | 1. Translation services; cultural or disability considerations? 2. Matching caregivers to appropriate volunteers/staff? 3. Adequate staffing or time to complete? 4. Adequate training of staff? 5. Is your practice authorized to bill for the time required to make referrals for peer support? 6. What other concerns do you have about the program/process? | **[OFFER]** | |
| 10. Are you aware of any champions or leadership support to improve the peer support referral process at your institution? Please describe. |  | **[OFFER]** | |
| 11. Are referrals to peer support programs something you think your practice should provide more of? | a. [If yes:] What would it take to encourage more referrals to peer support?  b. [If no:] Why not? | **[OFFER]** | |
| **[DO NOT OFFER]**  This section will identify those who have previously offered caregiver peer support referrals (PREV OFFERED) and those who have never offered referrals (NEVER OFFERED). Ask the appropriate questions based on the respondent’s answer.  **Organization’s or institution’s background** | | | |
| 1. Has your practice offered caregiver peer support referrals in the past? |  | **[DO NOT OFFER]** | |
| 2. What do you think are the challenges or barriers to offering peer support referrals? | 1. Translation services; cultural or disability considerations? 2. Matching caregivers to appropriate volunteers/staff? 3. Severity of disease/condition or length of treatment? 4. Inadequate staffing? Inadequate time to complete? 5. Inadequate training of staff? 6. Role of leaders and/or champions in peer support referrals? 7. Is your practice authorized to bill for the time required to make referral for peer support? | **[NEVER OFFERED]** | |
| 3. Why did your practice stop offering referrals to peer support? |  | **[PREV OFFERED]** | |
| 4. What were the major barriers to providing these referrals? | a. Translation services; cultural or disability considerations?  b. Matching caregivers to appropriate volunteers/staff?  c. Severity of disease/condition or length of treatment?  d. Inadequate staffing? Inadequate time to complete?  e. Inadequate training of staff?  f. Lack of familiarity with peer support referrals?  g. Lack of leaders and champions?  h. Lack of established internal peer support program? | **[PREV OFFERED]** | |
| 5. What was helpful in providing these referrals? |  | **[PREV OFFERED]** | |
| 6. Would you share any impacts these referrals may have had with families? | 1. Did it impact families’ stress level? 2. Did it impact families’ understanding of disease or condition? 3. Did families find peer support referral services valuable? | **[PREV OFFERED]** | |
| 7. Does your practice offer other services that help families navigate care for their children? If so, what kind of services? | a. Care navigators/therapy /Chaplin services?  b. Do you think referral to peer support services could supplement/complement or overlap with the services you already provided? | **[DO NOT OFFER]** | |
| 8. Have you received any feedback from families of children with special healthcare needs, regarding additional services they would like to see offered? | a. What services have they mentioned? | **[DO NOT OFFER]** | |
| 9. Do you view referrals to peer support as something your practice should offer? | a. Why or why not? | **[DO NOT OFFER]** | |
| 10. Are you aware of any champions or leaders at your practice who support adopting a peer support referral process? Please describe. |  | **[DO NOT OFFER]** | |
| 11. Would your practice consider offering referrals to peer support in the future? | 1. What would need to change for your practice to offer this type of referral? 2. What would help your practice implement a process to offer these referrals?   *i. Training?*  *ii. Introduction between practice and external peer support resources?*  *iii. Authorization to bill for time spent completing referral?* | **[DO NOT OFFER]** | |
| 12. Are there any other major barriers to implementing or concerns about peer support referrals at your practice? |  | **[DO NOT OFFER]** | |
| **Wrap-up** | | | |
| 1. Do you have any thoughts related to referrals to caregiver peer support that we have not addressed today? |  | **[ALL]** | |
| 2. Is there anyone else that you’d recommend we speak with about peer support referrals? | [If yes, collect contact information for this person.] | **[ALL]** | |
| That wraps up our questions for this interview. We will send you an email within the next couple of days with information about how to claim your $50 thank you payment, which you can receive as an Amazon gift card or as a donation to Special Olympics. Thank you so much for speaking with us today! | | | |
